# Transforming Disciplinary Traditions: Comment on "Problems and Promises of Health Technologies: The Role of Early Health Economic Modeling"

**DOI:** 10.15171/ijhpm.2019.130

**Published:** 2019-12-03

**Authors:** Pascale Lehoux, Hudson Silva

**Affiliations:** ^1^Department of Health Management, Evaluation and Policy, Institute of Public Health Research, University of Montreal, Montreal, QC, Canada.; ^2^Institute of Public Health Research, University of Montreal, Montreal, QC, Canada.

**Keywords:** Cost-Effectiveness, Health Innovation Value, System of Innovation, Priority Setting

## Abstract

Grutters et al show that economic assessments can inform the development of new health technologies at an early stage. This is an important contribution to health services and policy research, which implies a "shift away" from the more traditional forms of academic health economic modeling. Because transforming established disciplinary traditions is both valuable and demanding, we invite scholars to further the discussion on how the value of health innovations should be appraised in view of today’s societal challenges.

By showing that it is possible to inform at an early stage the development of new health technologies with cost-effectiveness analyses, the study of Grutters et al^1^ brings an important contribution to health services and policy research. According to the authors, one implication of their study emphasizes “a shift away from traditional use of health economic modeling” towards an analysis of “what is needed for a technology to provide most value for money.”^1^ In this commentary, we argue that what makes health innovations valuable in the context of 21st century societal challenges lies with dimensions that are broader than their costs and effects. We thus invite scholars to further the discussion on ways to transform our scholarly traditions.

## Informing Decisions That Are Made “Upstream” About Health Innovations

Aligned with previous work on the use of economic modeling in health technology assessment (HTA),^2^ one important key message from the study of Grutters et al^1^ is to recognize that health economists can provide innovators with precious insights at an early stage in the development of a new screening test, diagnostic or treatment. According to their study, such insights can help to redefine an innovation’s *value proposition* when it is still possible to do so, to inform how *further development* may enhance its cost-effectiveness or to *reposition* an innovation within the care pathway. Interestingly, up to 69% of the 32 early assessments these authors performed have led to the latter type of recommendations. It may reflect the knowledge gap that innovators sometimes face when they lack direct access to academic expertise in health services and policy research. Though innovators and their investors are familiar with market analyses, demographic projections and epidemiologic trends,^3^ they are less frequently exposed to findings regarding the comparative effectiveness of health interventions, the varying burdens of disease experienced by different patient sub-groups and current inefficiencies in care provision and their opportunity costs. These are precious insights that “untraditional” academic health economic research can provide.

We particularly appreciated the examples described by the authors since they illustrate very well how some of the key limitations of an innovation can be identified “upstream,” ie, before it makes its way into routine clinical practice.^4^ Stressing that an innovation’s cost-effectiveness is “always dependent on its context,” the authors recognize that “health economic modeling is only one tool to understand this context, and interviews with stakeholders to build and validate the model are essential.”^1^ In our view, it would prove equally important to consider the relationship between the various settings where an innovation may be used (academic centres, primary care centres, the patient’s home) and the level of skills required to use it appropriately since the latter influences overall costs (eg, training, user support, infrastructure, maintenance, etc).^4^

## Can Cost-Effectiveness Analyses Help Innovators Develop More Valuable Innovations?

We were however puzzled by the authors’ “unproblematized” use of the Dutch cost-effectiveness threshold of €20 000 to €80 000 per quality adjusted life year. If the aim is to shift away from scholarly traditions, questioning whether health economists should continue to use such thresholds seems warranted. The origin of the US$50 000 per quality adjusted life year benchmark that “first emerged in 1992 and became widely used after 1996” was loosely linked to the cost-effectiveness literature from the 1980s on kidney dialysis for end-stage renal disease, but the history of this practice and its implications for the sustainability of health systems are not always fully addressed.^5^ Without an explicit cost-effectiveness threshold, “there exists greater potential for decision-makers to feel increased pressure to approve or reject certain drugs due to the zeitgeist of the current political landscape.”^6^ Yet, if thresholds persist to raise as more expansive treatments continue to emerge in rich countries, they may not fulfill any meaningful purpose. As Griffiths et al^7^ underscore, “thresholds set so high that nearly all possible interventions are considered ‘cost‐effective’ cannot contribute effectively to priority setting.” Furthermore, critics consider that such thresholds “are unable to capture all the important values for society, particularly ethical implications, distributive justice, and other social preferences.”^8^

We also wonder whether such thresholds can guide innovators towards the development of the innovations contemporary health systems need. In other words, is it sufficient to assess costs and effects at an early stage and then seek to optimize the ratio? In their study, Grutters et al^1^ conclude that all of the 30 innovations they examined could potentially become cost-effective. This indirectly implies that they could all compete for the same limited healthcare resources. This is an issue the authors do not fully address, although they recognize the misalignment between a potentially cost-effective innovation and the administrative mechanism enabling its reimbursement, acquisition or use. Such barriers are well-known to health system observers, but they are not under the control of those who develop innovations. One may thus wonder whether early economic modelling may, in practice, guide the development of more valuable innovations.

For Grutters et al,^1^ other considerations such as “risks, competing upcoming innovations and logistical issues” may limit the commercial viability of an innovation even if it is potentially cost-effective. In view of the rising Research & Development costs and the high failure rate of emerging technology-based companies,^9^ we would argue in favor of making much more explicit these other considerations. Improving the efficiency of Research & Development processes in healthcare is a matter of high societal importance, one that goes beyond health system governance and raises questions about the kinds of health innovation our systems of innovation should deliver.

## What Makes Health Innovations Valuable in View of Current Societal Challenges?

Rather than seeking to estimate the “exact cost-effectiveness of a technology,” Grutters et al^1^ suggest that health economists should further explore “what is needed for a technology to provide most value for money.” Perhaps “value for money” is a term that deserves further definition for such an exploration to prove fruitful in the long run. In their scoping review on early HTA models, Fasterholdt et al^3^ identified 24 models, which they considered “immature” as they lacked clarity about what is meant by “early” and “value.” In our view, the term “early” should be defined in relation to the transformational impact an evaluation may have over an innovation, while recognising that the time and efforts needed to develop a medical device differ considerably from those needed to develop a drug. An assessment would be called early when it can be used to redefine an innovation’s initial value proposition and/or when it can make its position within the care pathway more valuable for health systems. In other words, an assessment arrives “late” when it can no longer transform what the innovation can achieve in practice. With respect to what is meant by “value,” since three quarters of the 24 early HTA models Fasterholdt et al^3^ identified only assess costs and effectiveness, efforts to develop early assessment frameworks should also clarify when a focus on cost-effectiveness should be maintained and when broader upstream considerations should be considered as well.

More fundamentally, the “shift away” Grutters et al^1^ evoke should start by asking, ‘what makes health innovations valuable in view of today’s societal challenges?’ To address the United Nation Sustainable Development Goals or the Horizon 2020 societal challenges, recent work around socially responsible innovation in health bring to the fore additional value dimensions such as the sustainability of health systems, the reduction of health inequalities and the protection of the environment.^10-12^ Our own efforts to define a framework for Responsible Innovation in Health builds on the interdisciplinary policy-oriented field of research called Responsible Research and Innovation, which recognizes that today’s societal challenges should steer the development of the next generation of innovations.^10^

The Responsible Innovation in Health framework posits that health innovations should not only prove effective and safe, but should also address the needs and challenges of health systems in an equitable and sustainable way. One of its five value domains brings to health economists’ attention the importance of developing high-performing products as well as affordable ones, which is best captured under the concept of “frugality.” It draws attention to the extent to which an innovation is designed to deliver greater value to more people by using fewer resources such as capital, materials, energy and labour time. Frugality may increase the economic value of a health innovation: (1) by increasing its affordability, which may result from optimized innovation production processes and/or lower maintenance needs; (2) by focusing on core functionalities and ease of use in order to meet the requirements of a larger number of users; and (3) by optimizing its performance level in order to maximize the fit between its characteristics and its context of use (eg, robustness if used in difficult climatic conditions, economies of scale if used in large centers, etc).^13^

Empirical research suggests that frugal innovations are better known in low-resource countries. For instance, a non-profit manufacturing company in India developed intraocular lens and ophthalmic drugs to treat non-communicable eye diseases (cataract, glaucoma, age-related macular degeneration, diabetic retinopathy) in marginalized people.^14^ Nonetheless, the concept of frugality can be applied anywhere to design low-cost, high quality solutions, including sophisticated technologies (eg, gene circuits on paper for identifying pathogens, robots made of cellphone parts).^15^ Prime et al^16^ examined the Arbutus Drill Cover System, which is a reusable double-layered surgical-grade cover that fully encloses a hardware drill and transforms it into a surgical grade drill. Developed by a team of Canadian and Ugandan engineers, surgeons, nurses, reprocessing staff and managers, it costs around £1500. If the Arbutus Drill Cover System were to be adopted in the British National Health System (replacing approximately 5000 drills at £23 000 each), it would generate a saving of 94%.^16^

Overall, in view of today’s societal challenges, seeking to transform disciplinary traditions may prove both valuable and necessary. As such, Grutters et al^1^ should be lauded. Because scholarly traditions are resistant to change, we invite scholars to further the discussion on ways to provide innovators with useful insights at an early stage.

## Ethical issues

Not applicable.

## Competing interests

Authors declare that they have no competing interests.

## Authors’ contributions

Both authors have critically revised this comment and approved the final version.

## Authors’ affiliations

^1^Department of Health Management, Evaluation and Policy, Institute of Public Health Research, University of Montreal, Montreal, QC, Canada. ^2^Institute of Public Health Research, University of Montreal, Montreal, QC, Canada.
